# Cutaneous basaloid squamous cell carcinoma in a captive blue-fronted amazon (*Amazona aestiva)* in Paraguay: gross description, histological and immunohistochemical findings

**DOI:** 10.29374/2527-2179.bjvm002425

**Published:** 2025-10-03

**Authors:** Joerg Richard Vetter, Leila Gertrudis Maidana, Thalita Evani Silva de Oliveira

**Affiliations:** 1 Departamento de Recursos Faunísticos y Medio Natural, Facultad de Ciencias Veterinarias (FCV), Universidad Nacional de Asunción (UNA), San Lorenzo, Paraguay.; 2 Departamento de Ciencias Patológicas, Facultad de Ciencias Veterinarias (FCV), Universidad Nacional de Asunción (UNA), San Lorenzo, Paraguay.; 3 Histopathological Diagnosis Department, VERUM Diagnóstico Animal Pathology, Hortolândia, Brazil.

**Keywords:** avian disease, dermatopathy, neoplasia, keratinocytes, psittacine, doença aviária, dermatopatia, neoplasia, queratinócitos, psitacídeo

## Abstract

Squamous cell carcinoma (SCC) is a malignant neoplasm that usually arises from the integument, and is reported uncommonly in pet birds. Basaloid SCC is a variant from the prototypic squamous cell carcinoma and is a biologically high grade tumor that shows a sharp predilection for the base of the tongue, the hypopharynx and the supraglottic larynx, manifesting deep and lateral invasion. We present the case of an *Amazon aestiva* from Paraguay with a basaloid SCC in the skin, of 8 months of evolution. Histologically, the findings were compatible with poorly differentiated carcinoma, and immunohistochemistry resulted positive for cytokeratin AE1/AE3 and 34βe12, and negative for BerEP4, vimentin and CK7, suggesting a basaloid variant of SCC. The owner reported that two weeks after the surgical removal of the mass, the animal was found dead and buried immediately, which is why post-mortem studies could not be carried out. In humans, basaloid SCC is associated with the upper aerodigestive tract but reports distant metastasis, even to the skin, suggesting a primary tumor elsewhere. To the authors best knowledge, this is the first report of basaloid squamous cell carcinoma in a bird.

## Resumo

O carcinoma de células escamosas (CCE) é uma neoplasia maligna que geralmente se origina do tegumento e é relatada de forma incomum em aves de estimação. O CCE basaloide é uma variante do carcinoma de células escamosas prototípico e é um tumor de alto grau biológico que mostra uma forte predileção pela base da língua, hipofaringe e laringe supraglótica, manifestando invasão profunda e lateral. Apresentamos o caso de um *Amazona aestiva* do Paraguai com um CCE basaloide na pele, com 8 meses de evolução. Histologicamente, os achados foram compatíveis com carcinoma pouco diferenciado, e a imuno-histoquímica resultou positiva para citoqueratina AE1/AE3 e 34βe12, e negativa para BerEP4, vimentina e CK7, sugerindo uma variante basaloide do CCE. O proprietário relatou que, duas semanas após a remoção cirúrgica da massa, o animal foi encontrado morto e enterrado imediatamente, razão pela qual não foi possível realizar estudos post-mortem. Em humanos, o CCE basaloide está associado ao trato aerodigestivo superior, mas há relatos de metástases distantes, até mesmo na pele, sugerindo um tumor primário em outro local. Para o melhor conhecimento dos autores, este é o primeiro relato de carcinoma basaloide de células escamosas em uma ave.

## Introduction

Squamous cell carcinoma (SCC) is a malignant neoplasm of epidermal cells in which the cells show differentiation to keratinocytes (Goldschmidt & Goldschmidt, 2017). These tumors are histologically composed of infiltrative nests and cords of moderately undifferentiated to poorly differentiated squamous cells (Reavill, 2004). Cutaneous SCCs appear as proliferative, irregular, broad-based or ulcer-like masses, which are usually aggressive, locally invasive, but rarely form metastases (Reavill, 2004). Common sites of cutaneous manifestation in birds include the wings, beak, and uropygial gland (Koski, 2002) and there is a tendency for these tumors to develop at sites of chronic irritation (Turrel et al., 1987). In mammals, prolonged exposure to ultraviolet light has been associated with the development of SCC (Goldschmidt & Goldschmidt, 2017), but this does not appear to be a factor in birds (Reavill, 2004). Definitive diagnosis requires cytological or histopathological evaluation (Koski, 2002).

Basaloid SCC is a variant from the prototypic squamous cell carcinoma, and is a biologically high grade tumor that, in human cases, shows a sharp predilection for the base of the tongue, the hypopharynx and the supraglottic larynx, manifesting deep and lateral invasion (Batsakis & El Naggar, 1989). This carcinoma is distinguished by at least two cytomorphologic components: the squamous carcinoma is usually well to moderately differentiated and invasive (being by the squamous or basaloid components, or both); and the basaloid component, which may not have a unity with either the squamous carcinoma component or the surface mucosa, but where it is present it is unequivocal (Batsakis & El Naggar, 1989). The basaloid cells show an often lobular growth pattern, but it can also be in cords or trabeculae, where hyperchromatism is imparted by small, often closely apposed cells and dark nuclei (Batsakis & El Naggar, 1989).

Immunohistochemical markers allow the differentiation between diverse cellular groups, such as epithelial and mesenchymal neoplasias. Vimentin is used for the mesenchymal group. On the other hand, cytokeratin (CK) is an important structural protein, widely used in epithelial cells (Cooper et al., 1985). AE1/AE3 is a pancytokeratin, a combination of high and low molecular weight cytokeratin, and they are constituents of the cytoskeleton of epithelial cells, staining positive in most carcinomas (Goldschmidt & Goldschmidt, 2017; Mauldin & Peters-Kennedy, 2016). Cytokeratin 34βe12 recognizes high-molecular-weight CK1, CK5, CK10 and CK14, and is used for the detection of epithelial basal cells (Ono et al., 2012). BER-EP4 is a monoclonal antibody that reacts against a group of transmembrane glycoproteins with 34kD and 39kD that are found on the surface and in the cytoplasm of epithelial cells, and is marked positive in a series of carcinomas and non-neoplastic epithelial cells, except hepatocytes, mesothelium and squamous epithelium (Latza et al., 1990).

Parrots (Aves: Psittaciformes) are very popular in the pet trade due to their bright colors, their sociability and the ability to imitate human voices (Mori et al., 2017). Amazon parrots (*Amazona* spp) are native to central and south America, but due to the growing pet trade they are now present all around the world, even establishing stable populations outside their native range, in Europe (Mori et al., 2017; Sciabarrasi et al., 2019). Although SCC is a relatively uncommon neoplasm in birds, mostly related to old animals, the most frequent reports are in different species of psittacine birds (Jones et al., 2020; Reavill, 2004; Sánchez-Godoy et al., 2020; Turrel et al., 1987).

The objective of this paper is to present a case of squamous cell carcinoma, basaloid variant, in a blue-fronted amazon (*Amazona aestiva*) in Paraguay, including the gross description, histopathologic and immunohistochemical findings.

## Case report

We describe the case of a captive blue-fronted amazon (*Amazona aestiva*), approximately 30 years old, unknown sex, 595 gram (g) body weight and low body condition (2/5) (Grespan & Freitas Raso, 2014). The animal had a history of chronic malnutrition due to inadequate feeding, evidenced also by darkened and neglected plumage. The owner kept the animal on a metal ring under a tree, exposed to the environment.

**Figure 1:**
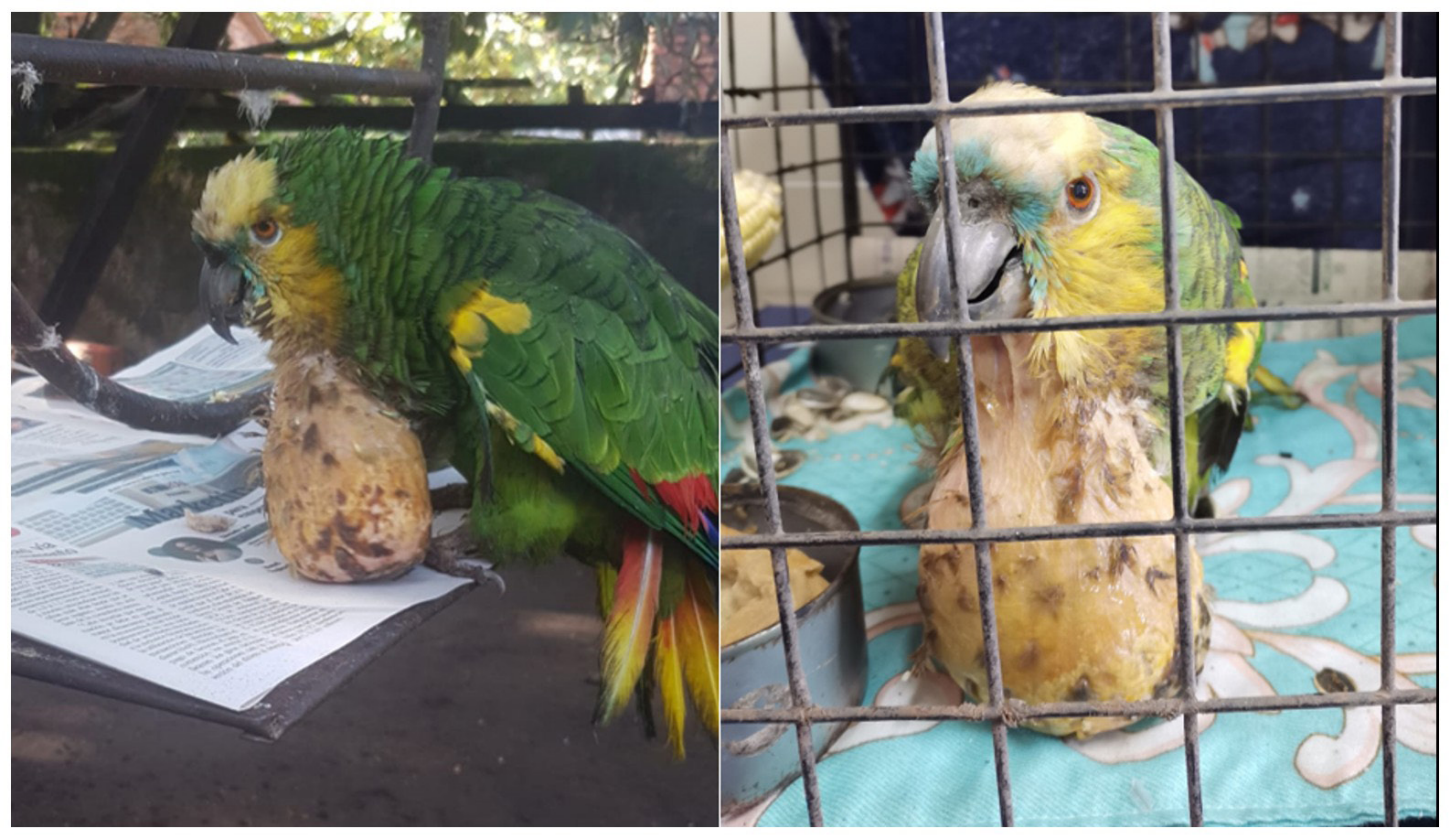
Macroscopic evaluation of mass in ventral neck region with feather loss and the affected skin displayed a slightly thickened, irregular epidermis.

On physical inspection, a mass was observed in the ventral neck region, approximately 8 cm in diameter, firm, mobile, with no increase in sensitivity, and with an approximate evolution of 8 months, according to the owner (Figure 1).

**Figure 2:**
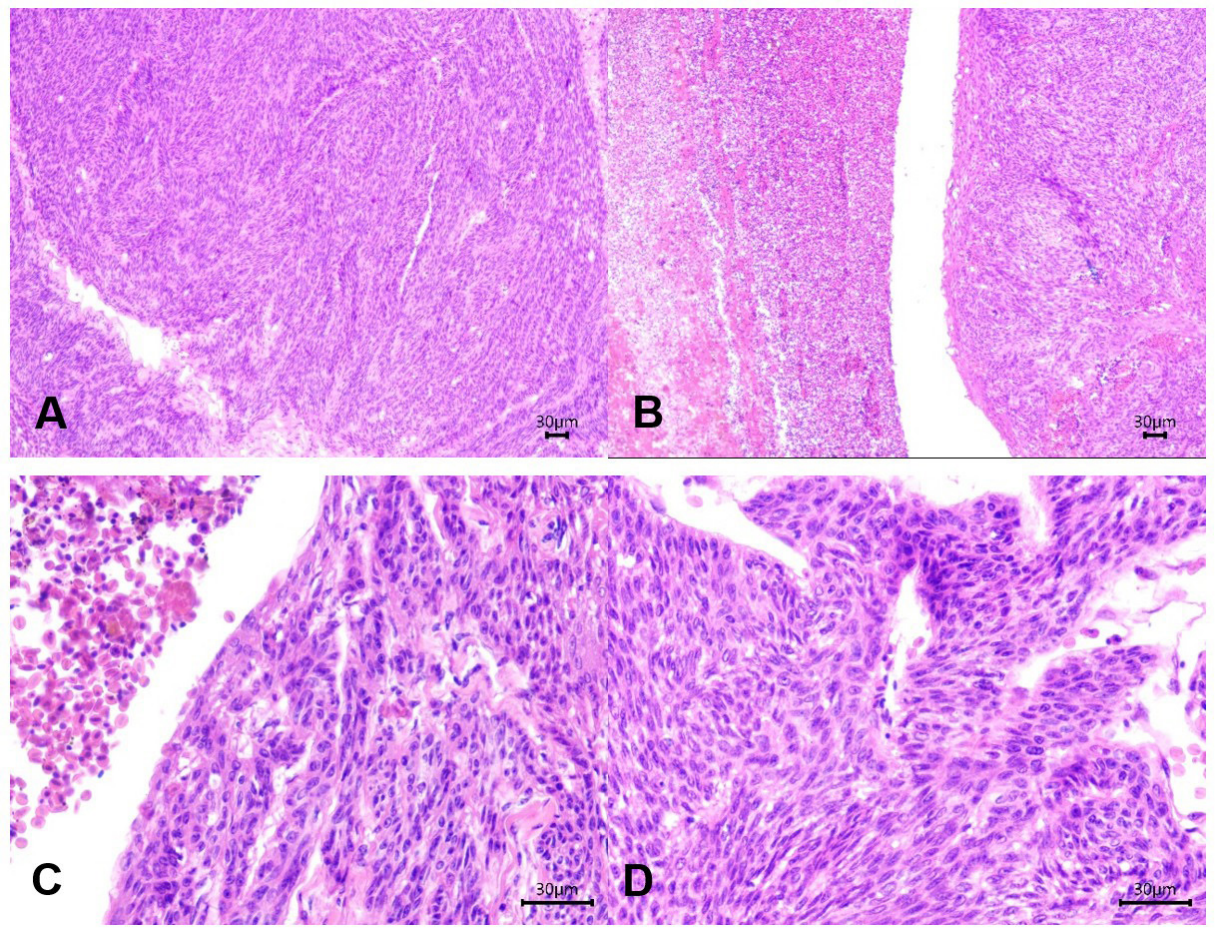
Cutaneous squamous cell carcinoma (SCC) basaloid variant in blue-fronted amazon (*Amazon aestiva*). (A) SCC comprised anastomosing cords and nests of neoplastic keratinocytes infiltrates, effaces, and replaces the dermis; (B, C) Neoplastic keratinocytes surround absent keratin pearls, along with heterophilic infiltrates and surrounded by marked fibrosis exfoliated squamous epithelium, necrotic debris, and degranulated heterophils; (D) Pleomorphic keratinocytes, with ovoid nuclei and 1-3 nucleoli. (A, B) H-E, 20x. (C, D) H-E, 40x. Bar: (A) to (D) 30 μm.

The mass was surgically removed and weighed 205 g. The surgical specimen was fixed in a 10% formaldehyde solution, dehydrated in increasing alcohols and embedded in paraffin for histological analysis, sectioned at 5µm thickness. The slides were stained with hematoxylin and eosin and microscopically showed epithelial tissue with neoplastic cell proliferation, organized in cords, infiltrative, made up of epithelial cells and endophytic and exophytic basal projection. The cells showed anisocytosis and moderate anisokaryosis, prominent round nucleus with reticular chromatin and single nucleolus, delimited cytoplasm of variable quantity and eosinophilic. Dermis with areas of hemorrhage and congestion. The findings were compatible with poorly differentiated carcinoma (Figure 2). The owner reported that two weeks after the surgical removal of the mass, the animal was found dead and buried immediately, which is why post-mortem studies could not be carried out.

Paraffin blocks were subjected to immunohistochemical techniques on skin sections for vimentin, pancytokeratin (AE1/AE3), Ber-EP4, cytokeratin 7 (CK7), and 34βe12 markers, as well as ki-67 cell proliferation. These were prepared on silanized slides with 0.1% poly-L-lysine (Sigma-Aldrich, St. Louis, MO, USA). The steps of IHC protocol are shown in Table 1.

**Table 1:** Characterization of the primary antibody and the antigen retrieval method.

**Primary Antibody**	**Clone**	**Dilution**	**Source**	**Antigen retrieval**	**pH**	**Method**	**Negative control**	**Positive control**
PanCk	AE1/AE3	1:300	Santa Cruz Biotechnology	Citrate	5.6	pressure cooker, 15 min	Absence of primary antibody	Dog skin
Ber-EP4	JCB117	1:150	Sigma-Aldrich	EDTA	8.6	pressure cooker, 3min	Absence of primary antibody	Dog lung adenocarcinoma
34βe12	M630	1:150	Dako	Citrate	5.6	pressure cooker, 15min	Absence of primary antibody	Dog thymus
Vimentin	V9	1:400	Dako	Citrate	5.6	Overnight	Absence of primary antibody	Dog vaginal leiomyoma
CK7	OV-TL 12/30	1:200	Dako	Citrate	5.6	Overnight	Absence of primary antibody	Dog urothelial carcinoma
Ki-67	Mib-1	1:400	Dako	EDTA	8.9	pressure cooker, 3min	Absence of primary antibody	Dog small intestine

The sections were deparaffinized in an oven at 65 °C for 2 hours, and the deparaffinization process continued with the immersion of the slides in two xylene changes. Then, the sections were hydrated in decreasing concentrations of ethyl alcohol and then in distilled water (10 baths). Antigen recovery process in accordance with the specificities of the primary antibody (Table 1) and were pre-treated with peroxide of 3.5% hydrogen at 25 °C for 10 minutes to block the endogenous peroxidase followed by immersion in phosphate-buffered saline (PBS - pH 7.4). All sections were covered and incubated with primary antibodies in a with constant humidity at 4 °C for 20 hours. After this period, detection was carried out with the LSAB® kit (Dako: Agilent Technologies, Inc., Glostrup, Denmark). The complex antigen-antibody was revealed using 3,3'-diaminobenzidine (DAB) (Dako: Agilent Technologies, Inc., Glostrup, Denmark) 2 min and sections were counterstained with Harris hematoxylin.

For evaluation of immunohistochemical expression by Ber-EP4, panCK, CK7, Vimentin, and 34βe12, expression was considered positive if at least one cell presented deposition of brown chromogen (DAB) on the membrane or cytoplasm of tumor cells. Considering this population of cells, the extent of the area expressing the antibody was classified as negative or positive. For nuclear proliferation markers, ki-67, the number of positive cells was evaluated by the total number of cells in each field, where 5 fields per 2,37mm^2^ area were evaluated under a 40x magnification. The result of this evaluation, the arithmetic mean, is the expression index.

**Figure 3:**
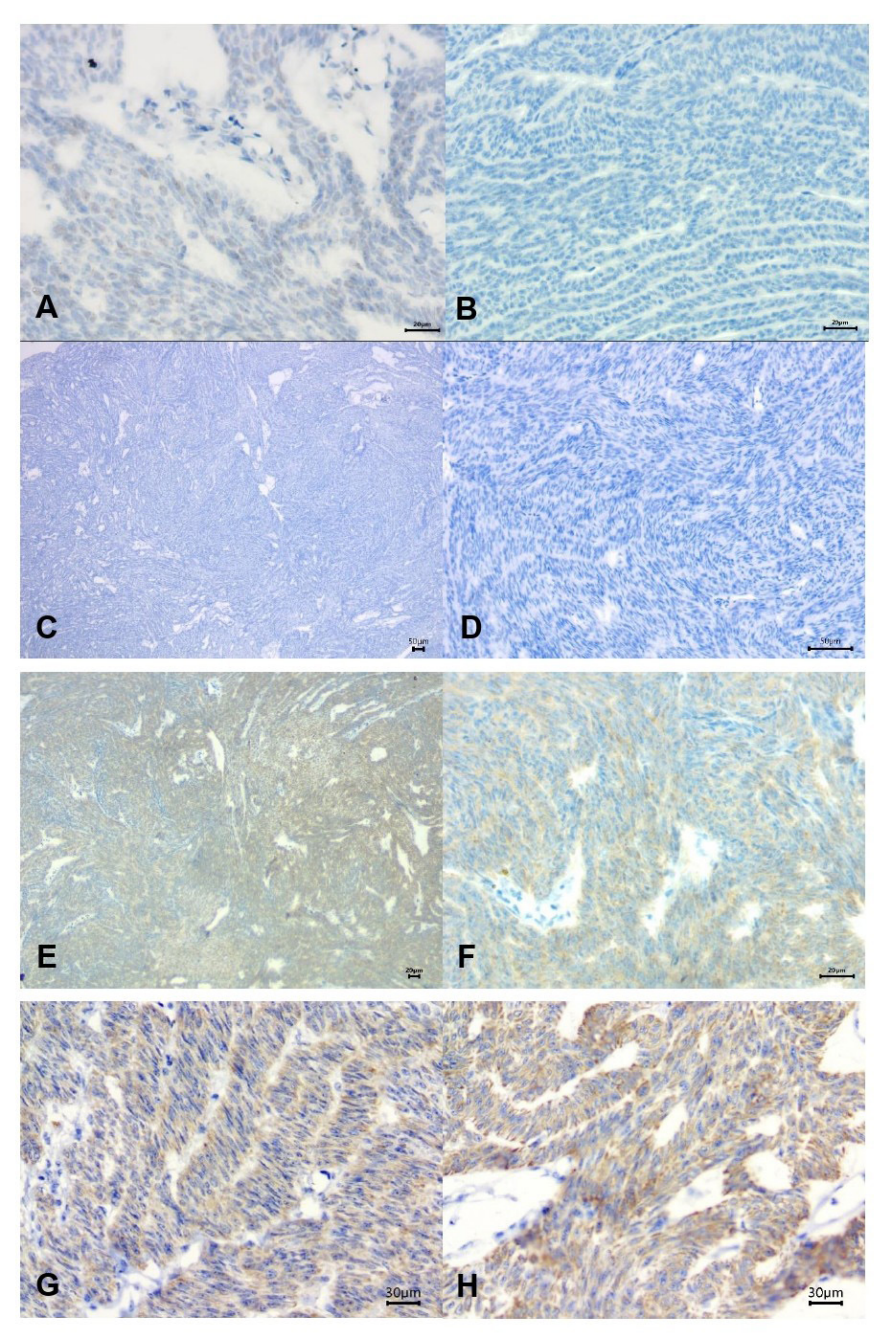
Expressed immunophenotyping panel in basaloid cutaneous squamous cell carcinoma in blue-fronted amazon (*Amazon aestiva*). (A) Ki67 staining was marked in around 20% of tumors cells; (B-D) Negative expression for basal cells (BerEP), mesenchymal cells (Vimentin), and squamous cells cytokeratin (CK7). Epithelial sample negative for the immunohistochemical markers; (E, F) Moderate intensity expression and intracytoplasmic staining for pancytokeratin. (AE1/AE3); (G, H) Strong perimembranous and cytoplasmatic labeling throughout for 34βe12. Immunoperoxidase counterstained with hematoxylin. Bar: (A, B, E, F) 20 μm; (C, D) 50 μm; (E, F) 30 μm.

All samples were negative to Ber-EP4, CK7 and vimentin expression (Figures 3AD), and positive for cytokeratin AE1/AE3 (Figures 3E, F) and 34βe12 (Figures 3G, H), with cytoplasmatic immunolabeling, which suggests a basaloid squamous cell carcinoma. Ki-67 proliferation index reported around 20% immunoreactivity

## Discussion

The general condition of the patient was consistent with chronic malnutrition in birds, which can disrupt or prolong the molting process. As a result, chronically malnourished birds often have dull and untidy plumage, with stress lines and/or changes in natural feather colouring, and thickened and scaly skin, especially on the face, legs, and around the cloaca (Koski, 2002).

While it is estimated that 90% of non-melanoma skin cancer is attributable to excessive exposure to UV radiation, which is described in several experimental reports in rats (Huang & Balmain, 2014), exposure to UV light does not appear to be a factor in SCC in pet birds because tumours occur at sites that are not usually exposed to prolonged sunlight (Reavill, 2004). Genetic factors, papillomaviruses and hypovitaminosis A may predispose to the development of SCC (Ginn et al., 2016; Zehnder et al., 2016). The influence of epidermal lesions on the onset of SCC has not yet been determined, but this theory is supported by the increased risk of squamous cell carcinoma in areas of ear notching, scarring, burns and chronic inflammation in mammals (Ginn et al., 2016). Basaloid SCC, first described as such by Wain et al. (1986), is an uncommon but aggressive neoplasm with a predilection for the upper aerodigestive tract, characterized by a high incidence of lymph node metastasis and distant spread, with reports including metastasis to the skin (Klijanienko et al., 1993; McKay & Bilous, 1989; Raslan et al., 1994; Shvili et al., 1990). The cutaneous manifestation in the present case could have been a primary tumor, or a skin metastasis, which would have been confirmed in a necropsy.

The use of immunohistochemical techniques is important when differentiating basal cell carcinoma from SCC, due to the differences in behaviour between the two tumours. BerEP4 staining is specific for BCC, so negative staining is suggestive of SCC (Beer et al., 2000; Robat et al., 2017; Sunjaya et al., 2017; Tell et al., 1997), although a case of BerEP4-negative BCC has been reported (Mleczko et al., 2011). The mixture of 2 different clones of anti-CK monoclonal antibodies (AE1 and AE3) functions as a broad-spectrum cytokeratin marker, where: AE1 detects the high molecular weight CK10, CK14, CK15 and CK16 as well as the low molecular weight CK19; and AE3 detects the high molecular weight CK1, CK2, CK3, CK4, CK5 and CK6 and the low molecular weight CK7 and CK8. This technique is not reactive to CK17 and CK18 (Lin et al., 2011). The CK AE1 / AE3 is a usual immunomarker as immunohistochemistry shows strong and diffuse membranous and cytoplasmic immunoreactivity in epithelial cells.

Vimentin is an intermediate filament, typically from cells of mesenchymal origin and non-epithelial tumors, and is used to determine the epithelial origin of histologically undifferentiated tumors, where mesenchymal tumor cells will stain positive, and epithelial tumor cells will stain negative (Amorim et al., 2016). Although sporadic expression of vimentin has been reported in SCC cells, this was related to less differentiated carcinomas and loss or reduction of cohesiveness (Liu et al., 2010; Liu et al., 2017). Basaloid SCC cases in humans, resembling the present case, have reported positive stains for 34βe12 and high weight CK, as well as negative stain for BerEp4, vimentin, among other CK, considering these to be helpful in the differential diagnosis with similar tumors (Andreadis et al., 2005; Boyd et al., 2011; Klijanienko et al., 1993; Tsang et al., 1991).

When the SCC develops in accessible areas, surgical excision is the treatment of choice (Reavill, 2004; Zehnder et al., 2016). Chemotherapy experiences mention complete regression with intralesional carboplatin treatment (Wilson et al., 2000), and topical 5-Fluorouracil (Filippich, 2004) although different treatments have had mixed success and more studies are needed (Robat et al., 2017).

As in other species, SCCs are resistant to radiation, making it difficult to treat larger or deeper lesions (Robat et al., 2017). Although radiotherapy has reported some partial responses in avian tumours (Freeman et al., 1999; Guthrie et al., 2010), a report on squamous cell carcinoma in *Ara ambigua* mentions that radiotherapy had no effect on the tumour (Manucy et al., 1998). It is suggested that birds may have a higher tissue tolerance to radiation and therefore probably require higher radiation doses (Barron et al., 2009; Manucy et al., 1998). This therapy requires more research in birds. Basaloid SCC prognosis is usually very poor and unpredictable when compared to conventional SCC (Altavilla et al., 1999).

## Conclusions

Immunohistochemistry allowed the differentiation of a histologically undifferentiated carcinoma, allowing the diagnosis of a rare neoplasm. To the authors best knowledge, this is the first report of basaloid squamous cell carcinoma in a bird. This report alerts clinicians and pathologists who work with birds to use immunophenotyping with standard markers for conventional pets, assisting in assertive diagnosis. Despite the local aggressiveness of the SCC, which makes complete resection difficult, an early diagnosis can translate into better treatment success, therapeutic and management approaches and patient prognosis.
